# Commentary: Why treatment is the best choice for childhood mental disorders – a commentary on Roest et al. (2022)

**DOI:** 10.1111/jcpp.13715

**Published:** 2022-11-02

**Authors:** Tycho J. Dekkers, Annabeth P. Groenman, Pim Cuijpers, Pieter J. Hoekstra, Marjolein Luman, Bram Orobio de Castro, Geertjan Overbeek, Arne Popma, Nanda Rommelse, Elske Salemink, Yvonne A.J. Stikkelbroek, Barbara J. van den Hoofdakker, Saskia van der Oord, Patty Leijten

**Affiliations:** ^1^ Accare Child Study Center Groningen The Netherlands; ^2^ Department of Child and Adolescent Psychiatry University Medical Center Groningen Groningen The Netherlands; ^3^ Levvel, Academic Center for Youth and Family Care Amsterdam The Netherlands; ^4^ Department of Child and Adolescent Psychiatry Amsterdam University Medical Centers Amsterdam The Netherlands; ^5^ Research Institute of Child Development and Education University of Amsterdam Amsterdam The Netherlands; ^6^ Department of Clinical, Neuro and Developmental Psychology, Amsterdam Public Health Research Institute Vrije Universiteit Amsterdam Amsterdam The Netherlands; ^7^ Karakter, Academic Center for Child and Adolescent Psychiatry Nijmegen The Netherlands; ^8^ Department of Psychiatry RadboudUMC Nijmegen The Netherlands; ^9^ Department of Clinical Psychology Utrecht University Utrecht The Netherlands; ^10^ Department of Clinical Child and Family Studies Utrecht University Utrecht The Netherlands; ^11^ GGZ Oost Brabant Boekel The Netherlands; ^12^ Department of Clinical Psychology and Experimental Psychopathology University of Groningen Groningen The Netherlands; ^13^ Clinical Psychology, Faculty of Psychology and Educational Sciences KU Leuven Leuven Belgium

**Keywords:** Child psychopathology, mental healthcare, long‐term effects, evidence‐based treatment

## Abstract

An important question in mental healthcare for children is whether treatments are effective and safe in the long run. Here, we comment on a recent editorial perspective by Roest et al. (2022), who argue, based on an overview of systematic reviews, ‘that there is no convincing evidence that interventions for the most common childhood disorders are beneficial in the long term’. We believe that the available evidence does not justify this conclusion and express our concern regarding the harmful effects of their message. We show that there is evidence to suggest beneficial longer term treatment effects for each of the disorders and explain why evidence‐based treatment should be offered to children with mental disorders.

## Abstract 2

Read the full article at doi: 10.1111/jcpp.13677.

## Introduction

With much interest we read the recent editorial perspective by Roest, de Vries, Wienen, and de Jonge (2022) who aimed to provide an overview of long‐term effects of treatment for children with mental disorders, published in the *Journal of Child Psychology and Psychiatry* on 29 August 2022. We appreciate the authors' discussion of a topic of large clinical relevance. However, we believe that the authors' conclusion ‘that there is no convincing evidence that interventions for the most common childhood disorders are beneficial in the long term’ is misguided.

The authors searched for systematic reviews of long‐term (in their definition over 2 years) outcomes of treatment for attention‐deficit/hyperactivity disorder (ADHD), behavioral disorders, anxiety, and depression for children between 6 and 12 years old. They identified five systematic reviews, all on ADHD or behavioral disorders, of which three focused on pharmacological treatments. We argue that the authors' conclusion is a classical misinterpretation of absence of evidence as evidence of absence. Moreover, the authors failed to discuss important findings that contradict their conclusion. We illustrate this by briefly reviewing the missed literature. As long‐term effects of healthcare are inherently multi‐factorial, and to a substantial degree influenced by non‐medical factors (i.e. societal, economic, and political), generalizing claims regarding the effects – or absence thereof – are hazardous and potentially harmful if policymakers use this to reduce the availability of mental healthcare services for the current generation of vulnerable children.

## State of the evidence

### Attention‐deficit/hyperactivity disorder (ADHD)

Evidence‐based clinical practice guidelines for the treatment of children with ADHD recommend behavioral parent training and/or stimulant medication. In addition to the numerous studies on short‐term benefits of these interventions (Daley et al., 2014), several studies suggest positive longer term outcomes of these interventions. Although many studies indeed did not follow children for more than 2 years, literature on longer term outcomes (based on 27 randomized controlled trials [RCTs]) of behavioral parent training for ADHD has recently been meta‐analyzed, pointing to sustained improvements of behavioral parent training on the longer term (Doffer et al., 2022). In addition, treatment with stimulant medication reduces the risk of many adverse health outcomes of ADHD on the long run, such as substance abuse, depression, suicide, accidental injuries, criminal activities, and teenage pregnancies, while adverse side effects are usually mild (see the World Federation of ADHD International Consensus Statement for 30 meta‐analyses or studies with at least 2,000 participants in favor of these claims; Faraone et al., 2021). Thus, for ADHD there is a clear indication that there are beneficial effects that exceed the immediate period after treatment.

### Behavioral disorders

Evidence‐based clinical practice guidelines for the treatment of children with behavioral disorders recommend behavioral parent training. The effect of parent training on children's behavioral problems is well established and numerous trials examined long‐term effects. The systematic review by Van Aar, Leijten, de Castro, and Overbeek (2017) identified 18 RCTs examining effects >1 year post intervention and 3 RCTs examining effects >2 year post intervention. In fact, some trials even followed children up to 5 or 10 years post intervention (Scott, Briskman, & O'Connor, 2014). The conclusions of these trials are consistent: on average treatment effects sustain, especially for children referred to treatment with more severe behavioral problems.

### Anxiety disorders

Evidence‐based clinical practice guidelines state that cognitive behavioral therapy (CBT) and selective serotonin reuptake inhibitor medication have considerable empirical support for treating children and adolescents with anxiety disorders (Walter et al., 2020). Perhaps surprisingly, Roest et al. identified no reviews for children with anxiety disorders. A meta‐analysis examined the durability of CBT effects, albeit not specifically for 6–12 years, but combined for children and adolescents (Rith‐Najarian et al., 2019). Aggregating information across broader age groups (beyond the 6–12 year range) is common practice in anxiety research since even an extensive individual patient data meta‐analysis showed no age effect in CBT response (Bennett et al., 2013). Rith‐Najarian et al. (2019) showed large (Hedges *g* = 2.79) within‐group long‐term effects (>2 years). This is consistent with an earlier review showing long‐term outcomes (average 5.85 years after initial CBT or medication treatment, range 2–19 years, Gibby, Casline, & Ginsburg, 2017). Thus, there are reviews and meta‐analyses examining the durability of treatment effects in the field of youth with anxiety disorders and these show sustained effects.

### Depression

All treatment guidelines recommend psychological treatments for children with depression as first‐line treatments, because of the importance and urgency of short‐term outcomes (Guideline Development Panel for the Treatment of Depressive Disorders, 2022; NICE Guideline NG134, 2019). Most studies focus on children and adolescents simultaneously and results are not separately examined. In one meta‐analysis, we found that the effects of psychotherapies for children and adolescents up to 2 years after baseline were still significant, although they were smaller in children and adolescents compared to adults (Cuijpers et al., 2020). One study in children, for example, found that after 2 years 80% did not meet criteria for depression anymore (Vostanis, Feehan, & Grattan, 1998). Thus, also for depression there is evidence to suggest beneficial long‐term effects.

## Why Roest et al. came to a misguided conclusion

There may be various reasons for why Roest et al. concluded that there are no positive longer term treatment effects. First, the absence of systematic reviews on long‐term effects of at least 2 years after treatment should not be confused with the absence of evidence of these effects. Evidence is provided by individual trials, and merely summarized in systematic reviews. Roest et al. ignored evidence provided in rigorous individual trials in all domains and have not included relevant review papers in specific domains (e.g. Shaw et al., 2012). For all conditions discussed above, evidence shows that treatment does have positive long‐term effects.

Second, any definition of ‘long‐term’ is arbitrary and the authors' choice for 2 years is no exception. Numerous studies (and various systematic reviews and meta‐analyses) show sustained treatment effects up to 12–18 months post intervention, suggesting that treatment effects are relatively stable over time. Given the current situation in which outcomes beyond 2 years have been less studied, information about any *longer*‐term effects (e.g. also 12 or 18 months post intervention) should weigh heavily in making treatment decisions. We appreciate that Roest et al. stress the methodological problems of examining long‐term outcomes of treatments in mental health for children, such as loss of randomization, variability in the natural course of mental disorders and general time‐related factors. Importantly, however, these problems should not be used as an argument against treatment. For example, the fields of adult psychosis and suicidality suffer from the same methodological problems in examining long‐term effects, but there is clear consensus about the importance of providing evidence‐based treatment.

Third, even if the current treatments would benefit families for ‘only’ 2 years, then 2 years of reduced suffering is valuable. Moreover, evidence‐based treatment reduces costs (e.g. less service use, higher parental productivity), as indicated by several cost‐effectiveness studies (e.g. Dijk et al., 2021; Posthumus, 2009). For example, when a dentist fills a cavity, no one expects this to be sufficient to prevent all possible dental issues later in a person's life. Yet, filling the cavity is highly effective and cost‐effective. We argue that the same holds for treatment for child mental health, where we should reduce suffering if we can.

Fourth, the authors express their concerns about the possible harms of treatment. What seems to be ignored here, is the harm of not providing treatment. Mental health problems in some children and youth fade over time, but the problems of others grow into chronic disorders with subsequent adverse outcomes, as also acknowledged by the authors. Decades of rigorous research provided us with numerous evidence‐based treatments that show these positively affect the lives of children and their families. It would not be just to withhold children from such support.

Last, Roest et al.'s umbrella review does not follow existing guidelines for umbrella reviews (e.g. AMSTAR‐2; Shea et al., 2017; Ioannidis, 2009). Many items on the AMSTAR‐2 checklist are not met by Roest et al. For example, the review was not registered, no deadlines were reported, it is not clear whether all data were extracted by independent researchers, and it was not clear how many records were screened or excluded. Also, the authors did not rate the quality of the systematic reviews they identified nor included such quality ratings in their assessments of the outcomes of the systematic reviews. These methodological limitations weaken the conclusions that can be drawn from Roest et al.'s review.

## Conclusion

There is sound evidence that interventions for children with mental disorders are effective, also on the longer term, and thus that treatment is the best choice for children with ADHD, behavioral disorders, anxiety, and depression. We believe this is an important message for clinicians, and a hopeful message for children and families in need of help.

## Conflicts of interest

T.J.D. was supported by an implementation grant of ZonMw (07290202110010). M.L. has co‐developed a self‐help teacher training program, without financial interests. She has received research grants from ZonMw and was an advisor of the Dutch ADHD guideline groups. P.J.H. has been a member of an advisory board meeting of Takeda. G.O. was supported by a grant from the Dutch Science Council (016.vici.185.063) in the preparation of this manuscript. E.S. was supported by a grant from the Dutch Science Council (VI.vidi.195.041) in the preparation of this manuscript. B.v.d.H. has received royalties as one of the editors of Sociaal Onhandig (published by Van Gorcum), a Dutch book for parents that can be used in parent training. She has been involved in the development and evaluation of several parent and teacher training programs, without financial interests; she has been a member of Dutch ADHD guideline and practice standard groups. S.v.d.O. has co‐developed a planning‐focused and solution‐focused treatment and other behavioral treatments but has no financial interest in any of these. She has received research grants from ZonMw and the Research Foundation Flanders (FWO); she was an advisor of the Dutch ADHD guideline groups and is a member of a working group on ADHD of the Superior Health Council of Belgium. P.L. was supported by a grant from the Dutch Science Council (VI.Vidi.201.065) in the preparation of this manuscript. None of the funders or other parties mentioned here had any involvement in the current commentary. The remaining authors have declared that they have no competing or potential conflicts of interest.
